# Threshold Effect of Cumulative Postnatal Corticosteroid Dose on Long-Term Outcomes in Extremely Preterm Infants

**DOI:** 10.3390/jcm15135023

**Published:** 2026-06-27

**Authors:** Na Hyun Lee, Ga Won Jeon, Soo Jeong Choo, Seung Hyun Kim, Hyeseon Kim, Misun Yang, So Yoon Ahn, Se In Sung, Yun Sil Chang

**Affiliations:** 1 Department of Pediatrics, Samsung Medical Center, Sungkyunkwan University School of Medicine, Seoul 06351, Republic of Korea; nahyun8613.lee@samsung.com (N.H.L.); soojeong.choo@samsung.com (S.J.C.); seunghyun2.kim@samsung.com (S.H.K.); hyeseon1.kim@samsung.com (H.K.); misun.yang@samsung.com (M.Y.); soyoon.ahn@samsung.com (S.Y.A.); sein.sung@samsung.com (S.I.S.); 2 Department of Pediatrics, Kangbuk Samsung Hospital, Sungkyunkwan University School of Medicine, Seoul 03181, Republic of Korea; iamgawon@hanmail.net; 3 Department of Health Sciences and Technology, Samsung Advanced Institute for Health Sciences & Technology (SAIHST), Samsung Medical Center, Seoul 06351, Republic of Korea

**Keywords:** bronchopulmonary dysplasia, corticosteroids, developmental disability, drug dose–response relationships, extremely preterm infants

## Abstract

**Background/Objectives**: To evaluate the association between cumulative dexamethasone dose (CDD) and long-term outcomes in extremely preterm infants (EPIs, gestational age <28 weeks). **Methods**: We retrospectively reviewed the medical records of 518 EPIs admitted from 2013 to 2021. Infants were categorized by CDD into five groups: no corticosteroids, <2 mg/kg, 2–<4 mg/kg (reference), 4–<8 mg/kg, and ≥8 mg/kg. Outcomes were assessed at corrected age of 18–24 months, including neurodevelopmental impairment (NDI), and failure to achieve catch-up growth. **Results**: Of 518 infants, 91.7% received postnatal corticosteroids. The median CDD was 4.24 mg/kg, initiated at a median age of 10 days. Among the 400 survivors at a corrected age of 18–24 months, 386 were followed up to evaluate long-term outcomes. A CDD ≥ 8 mg/kg was significantly associated with higher risks of NDI or death (adjusted OR 3.550, 95% CI: 1.260–10.004), NDI (adjusted OR 3.479, 95% CI: 1.074–11.268), and failure to achieve catch-up growth (adjusted OR 4.077, 95% CI: 1.217–13.656), compared to the reference group. No significant differences in NDI or failure to achieve catch-up growth were found in groups with CDD < 8 mg/kg. **Conclusions**: CDD ≥ 8 mg/kg was associated with significantly increased risks of adverse neurodevelopmental and growth outcomes, even when initiated after a median age of 7 days. Lower cumulative doses were not associated with increased risk, suggesting that, in critically ill infants with severe bronchopulmonary dysplasia (BPD), such doses may help reduce BPD severity and BPD-related mortality.

## 1. Introduction

Based on previous studies demonstrating that dexamethasone facilitates weaning from respiratory support and reduces the risk of bronchopulmonary dysplasia (BPD) in preterm infants, the use of corticosteroids in this population has become increasingly common. Due to their potent anti-inflammatory effects, postnatal corticosteroids may decrease the risk or severity of BPD—a major cause of morbidity among preterm infants and one of the leading causes of death and BPD-associated neurodevelopmental impairment (NDI) [1]. As survival among extremely preterm infants (EPIs, gestational age <28 weeks) has improved, the number of infants surviving with BPD has increased, leading to more frequent use of prolonged or high-dose corticosteroid therapy in this population. However, growing concern exists that corticosteroids may adversely affect neurodevelopmental outcomes, particularly by increasing the risk of cerebral palsy [2].

Regarding the timing of postnatal corticosteroid administration, there is a general consensus that early administration of dexamethasone—particularly within the first week of life—is discouraged, as it is associated with an increased risk of adverse neurodevelopmental outcomes, especially cerebral palsy [3,4,5,6]. With respect to indications, postnatal corticosteroids are discouraged in low-risk infants because the risk–benefit balance favors harm over benefit. Jensen et al. reported that postnatal corticosteroids reduced the risk of death or NDI in infants at high risk of death or grade 2 or 3 BPD, but may be harmful in those at lower risk [7]. Similarly, Doyle et al. found that postnatal dexamethasone increased the likelihood of survival without cerebral palsy in infants at high risk for BPD, but was detrimental in lower-risk infants [8]. Due to these concerns, several studies have aimed to develop treatment protocols based on respiratory severity to establish appropriate indications and minimize corticosteroid exposure in low-risk infants [9,10]. Nevertheless, postnatal corticosteroids are considered an indispensable therapy for severe BPD in EPIs. Therefore, a comprehensive understanding of postnatal corticosteroid therapy is essential. Although consensus has been reached regarding the timing and indications for postnatal corticosteroid use, limited research has examined the dose-dependent effects and threshold of the cumulative corticosteroid dose on long-term neurodevelopmental outcomes in EPIs, who are at the highest risk for both BPD and poor neurodevelopmental outcomes. Furthermore, there is a paucity of studies investigating the overall use of postnatal corticosteroids during hospitalization in EPIs and the associated outcomes according to the cumulative corticosteroid dose.

Accordingly, the aim of this study was to investigate the overall use of postnatal corticosteroids during hospitalization in EPIs and whether the cumulative corticosteroid dose is associated with long-term growth and developmental outcomes at a corrected age of 18–24 months, whether this association is dose-dependent, and whether a threshold dose exists.

## 2. Materials and Methods

### 2.1. Study Design and Population

**Figure 1:**
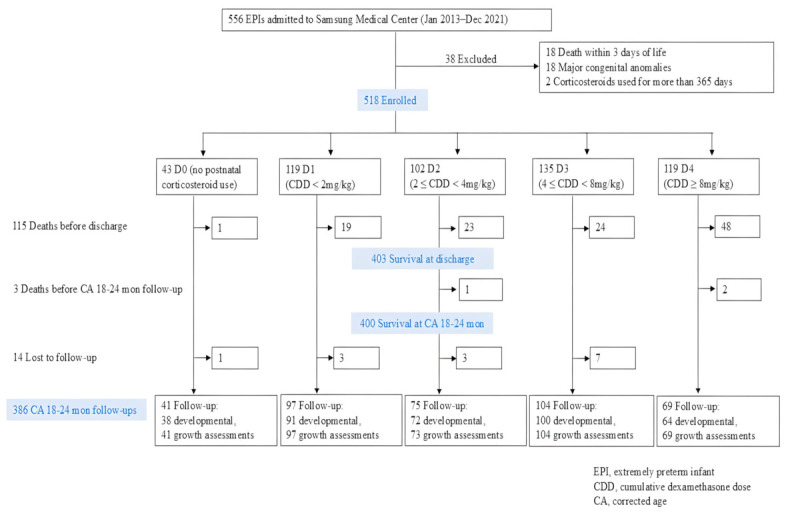
Study population and follow-up of cumulative dexamethasone dose group. A total of 556 extremely preterm infants (gestational age 22–27 weeks) were admitted to the neonatal intensive care unit between January 2013 and December 2021. Infants who died within the first 3 days of life (*n* = 18), had major congenital anomalies (*n* = 18), or received corticosteroids for more than 365 days (*n* = 2) were excluded, resulting in a final study population of 518 infants. Based on cumulative dexamethasone dose (CDD), infants were categorized into five groups: no postnatal corticosteroid use (D0, *n* = 43); CDD < 2 mg/kg (D1, low dose, *n* = 119); 2 ≤ CDD < 4 mg/kg (D2, moderate dose, *n* = 102); 4 ≤ CDD < 8 mg/kg (D3, high dose, *n* = 135); and CDD ≥ 8 mg/kg (D4, extremely high dose, *n* = 119). Among the enrolled infants, 115 died before discharge and 3 died after discharge. Of the remaining 400, 14 were lost to follow-up, and 386 were successfully followed up at a corrected age of 18–24 months.

A retrospective review of medical records was conducted for EPIs (gestational age 22–27 weeks) admitted to the neonatal intensive care unit (NICU) at Samsung Medical Center between January 2013 and December 2021 (*n* = 556). Infants who died within the first 3 days of life (*n* = 18), had major congenital anomalies (*n* = 18), or received corticosteroids for more than 365 days (*n* = 2) were excluded, resulting in a final study population of 518 infants (Figure 1).

### 2.2. Exposure

We evaluated the cumulative exposure to systemic corticosteroid therapy. Postnatal corticosteroids used in the NICU at Samsung Medical Center included hydrocortisone, prednisone, and dexamethasone. The cumulative dexamethasone dose (CDD) was calculated by converting the administered hydrocortisone and prednisolone into dexamethasone-equivalent doses based on their well-established glucocorticoid potencies and by summing the weight-adjusted doses (mg/kg) over the treatment period [11].

- For hydrocortisone, the dexamethasone-equivalent dose is calculated as: daily hydrocortisone dose per body weight (mg/kg/day) ÷ 20 × 0.75, and the values are summed across all administration days.
- For prednisolone, the dexamethasone-equivalent dose is calculated as: daily prednisolone dose per body weight (mg/kg/day) ÷ 5 × 0.75, and the values are summed across all administration days.
- For dexamethasone, the daily dexamethasone dose per body weight (mg/kg/day) is summed directly across all administration days.

The sum of these three values is defined as the CDD [11].

We analyzed CDD, a highly skewed variable, in categorical form to allow for easier clinical interpretation and comparison between groups. Based on the CDD, infants were categorized into five groups: no postnatal corticosteroid use (D0, *n* = 43); CDD < 2 mg/kg (D1, low dose, *n* = 119); 2 ≤ CDD < 4 mg/kg (D2, moderate dose, *n* = 102); 4 ≤ CDD < 8 mg/kg (D3, high dose, *n* = 135); and CDD ≥ 8 mg/kg (D4, extremely high dose, *n* = 119). The cutoff values of 2 and 4 mg/kg were adopted from previous studies [12], whereas the cutoff of 8 mg/kg used to distinguish the high-dose and extremely high-dose groups was determined by receiver operating characteristic curve analysis. The CDD cutoff value determined by the Youden index was 7.5 mg/kg. However, for clinical convenience, this value was rounded and set at 8 mg/kg. At a cutoff of 7.5 mg/kg, the sensitivity was 0.8750, the specificity was 0.8308, the Youden index was 1.7058, and the accuracy was 0.8328. Considering that a single course of dexamethasone therapy based on the unit’s protocol results in a total cumulative dose of 2.85 mg/kg, the moderate-dose group (D2) was used as reference for comparison (Figure 1).

Demographic factors included gestational age, birth weight, gender, Apgar scores, Clinical Risk Index for Babies (CRIB) II score, small for gestational age (birth weight below the 10th percentile of a population-specific birth weight for a given gestational age) [13], mode of delivery, prenatal corticosteroid therapy, maternal diabetes mellitus, maternal hypertension, histologically confirmed chorioamnionitis, premature rupture of membranes, and oligohydramnios.

Morbidities included intraventricular hemorrhage (grade ≥ 3), periventricular leukomalacia, retinopathy of prematurity (requiring laser therapy), respiratory distress syndrome, BPD (≥moderate severity based on the National Institute of Child Health and Human Development definition) [14], ventilatory support (combined duration of invasive and non-invasive ventilation), patent ductus arteriosus (requiring surgical ligation) [15], duration of parenteral nutrition, necrotizing enterocolitis (stage ≥ 2), culture-proven sepsis, and length of hospital stay.

### 2.3. Outcomes

The primary outcome was a composite outcome of NDI or death at a corrected age of 18–24 months. NDI was defined as any mental, motor, or social developmental delay. Mental developmental delay was defined as: (1) a Mental Developmental Index < 70 on the Bayley Scales of Infant and Toddler Development (BSID)-II; (2) a cognitive or language score < 70 on the BSID-III; or (3) a cognition or language score below the cutoff value on the Korean Developmental Screening Test (K-DST). Motor developmental delay was defined as: (1) a Psychomotor Developmental Index < 70 on the BSID-II; (2) a motor score < 70 on the BSID-III; or (3) a gross motor or fine motor score below the cutoff value on K-DST. Social developmental delay was defined as: (1) a social–emotional score or an adaptive behavior score < 70 on the BSID-III; or (2) a sociality or self-help score below the cutoff value on the K-DST [16]. K-DST is highly correlated with BSID-II or III; however, when BSID-II or III was performed, their results were prioritized [17,18]. For infants not assessed using BSID-II or III, K-DST results were used.

The secondary outcomes were blindness; hearing loss requiring hearing aids; cerebral palsy (Gross Motor Function Classification System score ≥ 2); NDI; and failure to achieve catch-up growth (z-scores < −1.28 [equivalent to <10th percentile] in weight, height, or head circumference according to corrected age, based on the World Health Organization Child Growth Standards [19]) at a corrected age of 18–24 months.

### 2.4. Statistical Analyses

Continuous variables were presented as median [interquartile range, IQR] to account for skewed distributions, and categorical variables were presented as number (%). Comparison among groups were performed by one-way ANOVA or Kruskal–Wallis test for continuous variables, and chi-square test or Fisher’s exact test for categorical variables.

The association between CDD groups (D0–D4) and primary and secondary outcomes was evaluated using multivariable logistic regression, with D2 as reference. Models were adjusted for covariates including gestational age at birth, 1 min Apgar score, intraventricular hemorrhage (grade ≥ 3), periventricular leukomalacia, retinopathy of prematurity requiring laser therapy, BPD (≥moderate), necrotizing enterocolitis (stage ≥ 2), duration of parenteral nutrition, culture-proven sepsis, small for gestational age, and length of hospital stay. In cases of sparse data, Firth’s penalized likelihood logistic regression was applied. Adjusted odds ratios (ORs) with 95% confidence intervals (CIs) were reported, and ORs were compared across dose groups using D2 as the reference. Bonferroni correction was applied to account for multiple pairwise comparisons. All statistical analyses were conducted using SAS version 9.4 (SAS Institute, Cary, NC, USA), and *p*-values < 0.05 were considered statistically significant.

## 3. Results

### 3.1. Baseline Characteristics by CDD Group

The median gestational age of the study population was 25 + 3 weeks [24 + 2, 26 + 5], and the median birth weight was 730 g [610, 900] (Table 1). As CDD increased, gestational age and birth weight significantly decreased (*p* < 0.0001). Higher CDD was also associated with lower Apgar scores and higher CRIB II scores (*p* < 0.0001). There were no significant differences among the groups in gender, mode of delivery, prenatal corticosteroid therapy, or maternal and obstetric factors.

### 3.2. Corticosteroid Exposure and Related Complications by CDD Group

Of the 518 infants included, 91.7% (475/518) received postnatal systemic corticosteroids. Among those who were treated, the CDD was 4.24 mg/kg [2.00, 8.06], with treatment initiated at a median postnatal age of 10 days [7, 14] and a treatment duration of 33 days [17, 56] (Table 2). The median age at initiation was 9 days [6, 13] for hydrocortisone, 18 days [14, 25] for dexamethasone, and 69.5 days [51, 90] for prednisolone. The duration of corticosteroid use increased significantly with higher CDD (D1, 17 days; D2, 21 days; D3, 36 days; D4, 61 days; *p* < 0.0001). The incidence of sepsis, gastrointestinal perforation, hypertension and hypertrophic cardiomyopathy increased with higher CDD. The subgroup analysis of postnatal corticosteroid use according to gestational age is presented in Supplementary Table S1.

### 3.3. Mortality and Neonatal Morbidities by CDD Group

Of the 518 infants included, 115 (22.2%) died before NICU discharge. Three additional infants died after NICU discharge, before the corrected age of 18–24 months. Mortality rate at discharge increased with higher CDD (D0: 2.33%; D1: 15.97%; D2: 22.55%; D3: 17.78%; D4: 40.34%; *p* < 0.0001) (Table 3). The incidence of intraventricular hemorrhage (grade ≥ 3) (*p* = 0.0241), periventricular leukomalacia (*p* < 0.0001), and retinopathy of prematurity requiring laser therapy (*p* < 0.0001) increased with higher CDD. The incidence of BPD (≥moderate) and the composite outcome of BPD (≥moderate) or death also increased with CDD (BPD: D0, 9.52%; D4, 93.41%; *p* < 0.0001; BPD or death: D0, 11.63%; D4, 94.96%; *p* < 0.0001). Total ventilatory support, duration of invasive ventilation, and duration of parenteral nutrition increased progressively with higher CDD (all *p* < 0.0001).

### 3.4. Long-Term Growth and Neurodevelopmental Outcomes by CDD Group

The follow-up rate at the corrected age of 18–24 months was 386 out of 400 (96.5%). The incidence of the composite outcome of NDI or death increased with higher CCD (D0: 16.67%; D4: 82.35%; *p* < 0.0001) (Table 4). The incidence of cerebral palsy also increased significantly with CCD, from 2.44% in D0 to 31.88% in D4 (*p* < 0.0001). NDI (including mental, motor, and social domains) and failure to achieve catch-up growth became more prevalent with increasing CCD (all *p* < 0.0001). This trend was also observed in each specific growth parameter—weight, height, and head circumference.

### 3.5. Associations of Long-Term Growth and Neurodevelopmental Outcomes by CDD Group

The risk of NDI or death was significantly higher in the D4 group (extremely high dose), with an adjusted OR of 3.550 (95% CI: 1.260–10.004), compared to the D2 group (moderate dose, reference) (Table 5). The other groups (D0, D1, D3) did not show significant differences in mortality risk compared to D2.

**Figure 2:**
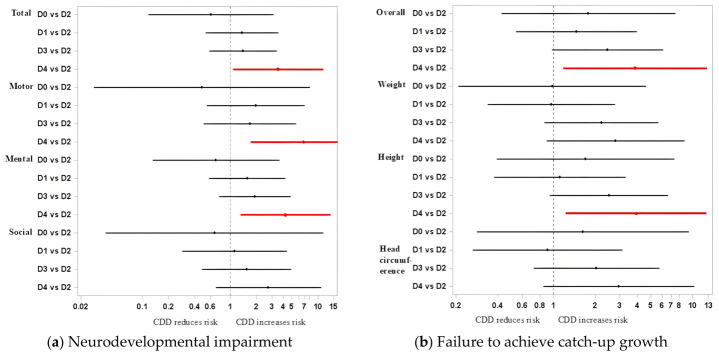
Associations of long-term outcomes by cumulative dexamethasone dose group. (**a**) The adjusted odds ratio (OR) of neurodevelopmental impairment is significantly increased in the D4 group (extremely high dose) compared to the D2 group (moderate dose, reference) (red line). In the mental and motor domains, the D4 group also shows a significantly increased adjusted OR compared to the D2 group (red line); (**b**) the adjusted OR of failure to achieve catch-up growth is significantly increased in the D4 group compared to the D2 group (red line). Specifically, the adjusted OR for failure to catch up in height is also significantly higher in the D4 group (red line).

The risk of cerebral palsy did not increase in the D4 group (OR: 1.587 [95% CI: 0.294–8.569]). The risk of NDI in the total domain was significantly increased in the D4 group, with an adjusted OR of 3.479 (95% CI: 1.074–11.268) (Table 5, Figure 2a). In the mental and motor domains, the D4 group also showed a significantly increased risk.

The overall risk of failure to achieve catch-up growth was significantly increased in the D4 group (OR: 4.077 [95% CI: 1.217–13.656]) (Table 5, Figure 2b). The risk of failure to catch up in height was also significantly higher in the D4 group.

Compared to the D2 group (reference), neither the D0 group (no corticosteroid use) nor the D1 group (low dose) showed a reduced risk of NDI or failure to achieve catch-up growth. Furthermore, the D3 group (high dose) did not show an increased risk of NDI or failure to achieve catch-up growth compared to the D2 group.

## 4. Discussion

In the present study, the median CDD was 4.24 mg/kg, and treatment was initiated at a median age of 10 days. A CDD ≥ 8 mg/kg (D4, extremely high dose) was associated with adverse long-term neurodevelopmental and growth outcomes. However, in the other groups with CDD < 8 mg/kg, no such associations were observed.

These findings suggest that when the CDD is kept below a certain threshold, postnatal corticosteroids may not adversely affect long-term neurodevelopmental and growth outcomes. Given that postnatal corticosteroids may be indispensable for treating life-threatening severe BPD, these findings could help alleviate clinicians’ concerns regarding their use. However, a 3.5-fold-higher risk of long-term NDI was observed when the CDD was ≥8 mg/kg, even with treatment initiated after a median age of 7 days. Aho et al. reported that a high CDD had long-term adverse effects on performance IQ at 6.5 years of age in EPIs, particularly when treatment was started earlier and the treatment course was longer [20].

According to a Cochrane systematic review, a CDD of ≥4 mg/kg was associated with increased risks of death or cerebral palsy, death or abnormal neurodevelopmental outcomes, and death compared to a dose of 2 ≤ CDD < 4 mg/kg [21]. In the present study, a CDD of ≥8 mg/kg was associated with increased risk of NDI and the composite outcome of NDI or death. In contrast to the Cochrane findings—where CDD ≥ 4 mg/kg was associated with adverse outcomes—no increased risk was observed in the 4 ≤ CDD < 8 mg/kg group. This discrepancy may be explained by the timing of corticosteroid administration. In the present study, the median age at initiation of postnatal corticosteroids was after the first week of life for hydrocortisone and after the second week for dexamethasone. This represents the median timing rather than indicating that all infants received treatment after these time points. It is hypothesized that the threshold CDD for inducing NDI may have been higher in this context, as corticosteroids were not administered during the early postnatal period, when the developing brain is believed to be more vulnerable. This hypothesis is supported by findings that late systemic dexamethasone (after a median age of 7 days) reduces the risk of death or BPD without increasing the risk of cerebral palsy [6,22], and also reduces the overall risk of death and BPD [12].

The mineralocorticoid activity of synthetic steroids protects against neuronal injury, whereas their glucocorticoid activity inhibits neuronal growth, induces apoptosis, and delays myelination [23]. Dexamethasone, which has only glucocorticoid effects, can reduce cerebral tissue volume [24], and early postnatal use (within the first week of life) increases NDI risk [3,4,5,6]. Hydrocortisone has mild glucocorticoid and mineralocorticoid activity [25], resulting in lower neurotoxicity; unlike dexamethasone, early postnatal use is not linked to cerebral palsy or NDI [5].

BPD is associated with white-matter abnormalities, delayed cortical maturation, and impaired neurodevelopment [26,27]. In high-risk infants, a 10-day low-dose dexamethasone course initiated after 7 days improves brain volumes and BSID-III motor scores [28], likely mediated by a reduced risk of BPD and its associated NDI. Cochrane reviews show no difference in cerebral palsy, death or cerebral palsy, and impaired neurodevelopment between low (<2 mg/kg) and moderate (2–4 mg/kg) CDD [21], consistent with our findings. However, higher cumulative corticosteroid dose was associated with an increased risk of cerebral palsy (adjusted OR 1.47) among EPIs [29].

Marr et al. reported that in high-risk EPIs, a 42-day dexamethasone regimen (total 7.56 mg/kg) improved ventilator duration and school-age neurodevelopment compared to a rapid 9-day taper (4.04 mg/kg) [30]. However, treatment longer than 14 days was associated with worse neurodevelopment [31]. While Marr’s study suggests some alleviation of concerns about minimizing corticosteroid exposure, opposing evidence exists, and corticosteroid use remains inconclusive [32].

The finding that a CDD ≥ 8 mg/kg was associated with a 4-fold increased risk of failure to achieve catch-up growth in the present study is consistent with previous reports of growth failure following postnatal corticosteroid use [33,34]. EPIs with BPD are at high risk for growth impairment, and corticosteroids may further exacerbate this risk [35]; therefore, they should be used with caution, particularly at higher cumulative doses. 

Infants with a CDD ≥ 8 mg/kg had lower gestational age and birth weight. To account for the potential confounding effects on long-term neurodevelopment and growth outcomes, adjustments were made for multiple baseline characteristics and neonatal morbidities, as described in the Methods section. Even after adjusting for all these variables, a CDD ≥ 8 mg/kg remained significantly associated with poor long-term neurodevelopmental and growth outcomes. Furthermore, despite the D0 group (no postnatal corticosteroid exposure) having higher gestational age and birth weight and lower neonatal morbidity, there was no reduction in the NDI, or failure to achieve catch-up growth. These findings suggest that the poor neurodevelopmental and growth outcomes observed in the CDD ≥ 8 mg/kg group are likely attributable to the high-dose corticosteroid.

This study has several limitations. First, the limitations include the inability to analyze outcomes according to individual corticosteroid types—hydrocortisone, dexamethasone, and prednisolone—as well as the lack of analysis based on the postnatal age at initiation and the duration of corticosteroid use. Second, infants in the D4 group had lower gestational age and birth weight. The initiation of corticosteroids was based on each infant’s clinical condition; thus, it is not surprising that infants treated with corticosteroids were sicker, which is an inherent limitation of retrospective studies. Although we adjusted for multiple confounding factors to account for their potential impact on long-term developmental and growth outcomes and to minimize the association between morbidities and CDD, residual confounding could not be completely eliminated in this retrospective study. In addition, because long-term neurodevelopmental outcomes are influenced by multiple factors, it cannot be concluded from this retrospective study that CDD alone was entirely responsible for the long-term neurodevelopmental outcomes in EPIs, who are at the highest risk for poor neurodevelopmental outcomes. A large-scale prospective study is needed to further evaluate the association between CDD and outcomes. Nevertheless, the strength of this study lies in its dose-dependent evaluation of short-term outcomes and long-term neurodevelopmental and growth outcomes according to the cumulative dose of postnatal systemic corticosteroids administered for various indications in EPIs. These findings may serve as a valuable reference for establishing corticosteroid treatment protocols and predicting long-term outcomes in this high-risk population.

## 5. Conclusions

CDD ≥ 8 mg/kg was significantly associated with adverse long-term outcomes, even when treatment was initiated after a median age of 7 days. However, lower cumulative doses were not associated with increased risks, suggesting that, in critically ill infants with severe BPD, such doses may help reduce BPD severity and BPD-related mortality, and potentially decrease BPD-associated NDI. These findings highlight the importance of cumulative dose-dependent caution when prescribing postnatal corticosteroids and may guide clinicians in optimizing treatment strategies for infants at high risk of BPD while minimizing long-term adverse effects.

## Figures and Tables

**Table 1:** Baseline characteristics by CDD group.

Variables	Total (*n* = 518)	D0 (No Postnatal Corticosteroid Use, *n* = 43)	D1 (CDD < 2 mg/kg, *n* = 119)	D2 (2 ≤ CDD < 4 mg/kg, *n* = 102)	D3 (4 ≤ CDD < 8 mg/kg, *n* = 135)	D4 (CDD ≥ 8 mg/kg, *n* = 119)	*p*-Value
Gestational age, weeks	25^+3^ [24^+2^, 26^+5^]	27^+1^ [26^+6^, 27^+4^]	26^+1^ [25^+2^, 27^+0^]	25^+2^ [24^+3^, 26^+4^]	25^+1^ [24^+1^, 26^+0^]	24^+4^ [23^+4^, 25^+4^]	<0.0001
Birth weight, g	730 [610, 900]	1000 [870, 1080]	830 [640, 940]	730 [630, 900]	690 [590, 810]	640 [640, 760]	<0.0001
Male	270 (52.12)	22 (51.16)	55 (46.22)	49 (48.04)	77 (57.04)	67 (56.3)	0.3426
Apgar score, 1 min	5 [4, 6]	6 [5, 7]	5 [4, 6]	5 [4, 6]	4 [3, 5]	4 [4, 5]	<0.0001
Apgar score, 5 min	8 [7, 8]	8 [8, 9]	8 [7, 8]	8 [7, 8]	8 [6, 8]	7 [6, 8]	<0.0001
CRIB-II score	13 [10, 14]	9 [9, 10]	11 [9, 13]	12 [10, 14]	13 [12, 15]	14 [13, 16]	<0.0001
Small for gestational age	100 (19.31)	3 (6.98)	22 (18.49)	14 (13.73)	31 (22.96)	30 (25.21)	0.0386
Cesarean section	415 (80.12)	34 (79.07)	96 (80.67)	80 (78.43)	110 (81.48)	95 (79.83)	0.9826
In vitro fertilization	169 (32.63)	12 (27.91)	34 (28.57)	36 (35.29)	42 (31.11)	45 (37.82)	0.5161
Multiple pregnancy	183 (35.33)	15 (34.88)	39 (32.77)	37 (36.27)	41 (30.37)	51 (42.86)	0.3097
Prenatal corticosteroid	496 (95.75)	43 (100)	111 (93.28)	97 (95.10)	130 (96.30)	115 (96.63)	0.3879
Maternal diabetes mellitus	29 (5.60)	5 (11.63)	10 (8.4)	2 (1.96)	8 (5.93)	4 (3.36)	0.0768
Maternal hypertension	76 (14.67)	4 (9.3)	24 (20.17)	12 (11.76)	25 (18.52)	11 (9.24)	0.0624
Chorioamnionitis	304 (58.68)	24 (55.81)	59 (49.58)	63 (61.76)	82 (60.74)	76 (63.87)	0.1870
Premature rupture of membrane	202 (39.00)	16 (37.21)	41 (34.45)	38 (37.25)	51 (37.78)	56 (47.06)	0.3360
Oligohydramnios	126 (24.32)	14 (32.56)	29 (24.37)	24 (23.53)	27 (20)	32 (26.89)	0.4908

CDD, cumulative dexamethasone dose; CRIB-II, clinical risk index for babies II. Values are expressed as median [IQR] or number (%). *p*-values are calculated by one-way ANOVA or Kruskal–Wallis test for continuous variables and Chi-square test or Fisher’s exact test for categorical variables, depending on data distribution and sparsity.

**Table 2:** Corticosteroid exposure and related complications by CDD group.

Variables	Total (*n* = 475)	D1 (CDD < 2 mg/kg, *n* = 119)	D2 (2 ≤ CDD < 4 mg/kg, *n* = 102)	D3 (4 ≤ CDD < 8 mg/kg, *n* = 135)	D4 (CDD ≥ 8 mg/kg, *n* = 119)	*p*-Value
Type of Corticosteroid						
Hydrocortisone	413 (86.95)	105 (88.24)	79 (77.45)	120 (88.89)	109 (91.6)	0.0064
Dexamethasone	312(65.68)	41 (34.45)	56 (54.9)	107 (79.26)	108 (90.76)	<0.0001
Prednisolone	125 (26.32)	4 (3.36)	20 (19.61)	47 (34.81)	54 (45.38)	<0.0001
Time of Initiation, Postnatal Day						
Total postnatal corticosteroids	10 [7, 14]	11 [7, 15]	10 [7, 15]	10 [5, 15]	9 [7, 12]	0.1359
Hydrocortisone	9 [6, 13]	10 [7, 15]	10 [6, 13]	9 [4, 14.5]	9 [6, 12]	0.5044
Dexamethasone	18 [14, 25]	18 [14, 24]	19 [14, 23]	18.5 [14, 24.5]	17.5 [13, 26.5]	0.9928
Prednisolone	69.5 [51, 90]	31 [26, 58]	52.5 [43, 63]	64 [53, 81]	81 [62, 110]	0.0001
Duration of Corticosteroid Use, Days						
Total postnatal corticosteroids	33 [17, 56]	17 [8, 31]	21 [11, 42]	36 [24, 57]	61 [43, 97]	<0.0001
Hydrocortisone	21 [10, 42]	17 [6, 31]	19 [8, 42]	18 [10, 43]	31 [14, 58.5]	<0.0001
Dexamethasone	11 [5, 22]	5.5 [0, 8]	8 [2, 13]	13 [8, 20]	26 [11, 36]	<0.0001
Prednisolone	0 [0, 12]	0 [0, 0]	0 [0, 10]	5.5 [0, 12]	11 [0, 22]	<0.0001
CDD, mg/kg	4.24 [2.00, 8.06]	1.05 [0.53, 1.44]	2.89 [2.4, 3.54]	5.49 [4.7, 6.88]	12.35 [10, 19.01]	<0.0001
Cumulative hydrocortisone, mg/kg (*n* = 413)	37.99 [15.62, 85.44]	18.65 [9.2, 29.47]	48.85 [23.47, 71.43]	42.41 [15.64, 88.03]	133.95 [42.57, 227.46]	<0.0001
Cumulative dexamethasone, mg/kg (*n* = 312)	3.42 [1.24, 6.2]	0.89 [0.82, 1]	2.36 [1.16, 3.16]	3.67 [2.05, 4.65]	7.38 [4.63, 11.26]	<0.0001
Cumulative prednisolone, mg/kg (*n* = 125)	16.68 [14.38, 27.96]	2.05 [0.23, 7.14]	14.59 [11.51, 16]	16.54 [14.34, 22.61]	25.02 [16.32, 36.56]	<0.0001
CDD per day, mg/kg/day	0.15 [0.07, 0.23]	0.05 [0.03, 0.11]	0.16 [0.07, 0.24]	0.16 [0.1, 0.23]	0.21 [0.16, 0.31]	<0.0001
Complications During Corticosteroid Use						
Culture-proven sepsis	91 (19.16)	15 (12.61)	10 (9.80)	21 (15.56)	45 (37.82)	<0.0001
Gastrointestinal perforation	57 (12.00)	6 (5.04)	9 (8.82)	16 (11.85)	26 (21.85)	0.0005
Hypertension	84 (17.68)	6 (5.04)	14 (13.73)	17 (12.59)	47 (39.50)	<0.0001

CDD, cumulative dexamethasone dose; CRIB-II, clinical risk index for babies II. Values are expressed as median [IQR] or number (%). *p*-values are calculated by one-way ANOVA or Kruskal–Wallis test for continuous variables and Chi-square test or Fisher’s exact test for categorical variables, depending on data distribution and sparsity.

**Table 3:** Mortality and neonatal morbidities by CDD group.

Variables	Total (*n* = 518)	D0 (No Postnatal Corticosteroid Use, *n* = 43)	D1 (CDD < 2 mg/kg, *n* = 119)	D2 (2 ≤ CDD < 4 mg/kg, *n* = 102)	D3 (4 ≤ CDD < 8 mg/kg, *n* = 135)	D4 (CDD ≥ 8 mg/kg, *n* = 119)	*p*-Value
Death before discharge	115 (22.20)	1 (2.33)	19 (15.97)	23 (22.55)	24 (17.78)	48 (40.34)	<0.0001
IVH (grade ≥ 3)	58/511 (11.35)	1 (2.33)	9/114 (7.89)	12/101 (11.88)	14 (10.37)	22/118 (18.64)	0.0241
PVL	38/511 (7.44)	0 (0)	3/114 (2.63)	1/101 (0.99)	11 (8.15)	23/118 (19.49)	<0.0001
ROP requiring laser therapy	80/408 (19.61)	0/32 (0)	9/100 (9.0)	7/72 (9.72)	24/110 (21.82)	40/94 (42.55)	<0.0001
RDS	501 (96.72)	34 (79.07)	118 (99.16)	98 (96.08)	134 (99.26)	117 (98.32)	<0.0001
BPD (≥moderate)	234/424 (55.19)	4/42 (9.52)	30/100 (30.0)	37/79 (46.84)	78/112 (69.64)	85/91 (93.41)	<0.0001
BPD (≥moderate) or death	328 (63.32)	5 (11.63)	49 (41.18)	60 (58.82)	101 (74.81)	113 (94.96)	<0.0001
Total ventilatory support, days	56 [30, 81]	24 [7, 32]	41 [22, 56]	52.5 [26, 69]	67 [43, 85]	98 [69, 139]	<0.0001
Duration of invasive ventilation, days	26 [9, 47]	1 [0, 2]	9 [3, 19]	22.5 [11, 30]	32 [22, 49]	56 [39, 82]	<0.0001
PDA (requiring surgical ligation),	8 (1.54)	0 (0)	0 (0)	1 (0.98)	2 (1.48)	5 (4.20)	0.0817
Time to enteral feeding ≥ 100 mL/kg/day, postnatal day	32 [20, 55]	13 [8, 22]	28 [18, 44]	33 [18, 54]	37 [24.5, 62]	49 [28.5, 74]	<0.0001
Duration of PN, days	39 [23, 64]	16 [12, 26]	33 [20, 50]	35 [21, 63]	47 [28, 67]	61 [35, 111]	<0.0001
NEC (stage ≥ 2)	93 (17.95)	1 (2.33)	22 (18.49)	14 (13.73)	28 (20.74)	28 (23.53)	0.0205
Culture-proven sepsis	126 (24.32)	2 (4.65)	23 (19.33)	15 (14.71)	30 (22.22)	56 (47.06)	<0.0001
Length of hospital stay	104 [74, 133]	75 [68, 81]	97 [73, 107]	100.5 [75, 123]	121 [91, 138]	140 [93, 202]	<0.0001

CDD, cumulative dexamethasone dose; IVH, intraventricular hemorrhage; PVL, periventricular leukomalacia; ROP, retinopathy or prematurity; RDS, respiratory distress syndrome; BPD, bronchopulmonary dysplasia; PDA, patent ductus arteriosus; PN, parenteral nutrition; NEC, necrotizing enterocolitis. Values are expressed as median [IQR] or number (%). *p*-values are calculated by one-way ANOVA or Kruskal–Wallis test for continuous variables and Chi-square test or Fisher’s exact test for categorical variables, depending on data distribution and sparsity.

**Table 4:** Long-term growth and neurodevelopmental outcomes at corrected age of 18–24 months by CDD group.

Variables	Total (*n* = 518)	D0 (No Postnatal Corticosteroid Use, *n* = 43)	D1 (CDD < 2 mg/kg, *n* = 119)	D2 (2 ≤ CDD < 4 mg/kg, *n* = 102)	D3 (4 ≤ CDD < 8 mg/kg, *n* = 135)	D4 (CDD ≥ 8 mg/kg, *n* = 119)	*p*-Value
Primary Outcome							
NDI or death	273/504 (54.17)	7/42 (16.67)	50/116 (43.10)	49/99 (49.49)	69/128 (53.91)	98/119 (82.35)	<0.0001
Secondary Outcome	Total (*n* = 386)	D0 (*n* = 41)	D1 (*n* = 97)	D2 (*n* = 75)	D3 (*n* = 104)	D4 (*n* = 69)	*p*-value
Blindness	1 (0.26)	0 (0)	0 (0)	0 (0)	0 (0)	1 (1.45)	0.2850
Hearing loss	13 (3.37)	1 (2.44)	3 (3.09)	0 (0)	4 (3.85)	5 (7.25)	0.4716
Cerebral palsy	49 (12.69)	1 (2.44)	3 (3.09)	7 (9.33)	16 (15.38)	22 (31.88)	<0.0001
NDI	155/365 (42.47)	6/38 (15.79)	31/91 (34.07)	25/72 (34.72)	45/100 (45)	48/64 (75.00)	<0.0001
Motor delay	89/365 (24.38)	3/38 (7.89)	17/91 (18.68)	10/72 (13.89)	21/100 (21)	38/64 (59.38)	<0.0001
Mental delay	120/365 (32.88)	4/38 (10.53)	26/91 (28.57)	17/72 (23.61)	35/100 (35)	38/64 (59.38)	<0.0001
Social delay	66/341 (19.35)	1/34 (2.94)	9/86 (10.47)	9/69 (13.04)	20/94 (21.28)	27/58 (46.55)	<0.0001
Failure to achieve catch-up growth (<10th percentile)	162/384 (42.19)	10/41 (24.39)	30/97 (30.93)	20/73 (27.40)	53/104 (50.96)	49/69 (71.01)	<0.0001
Weight	116/384 (30.21)	6/41 (14.63)	19/97 (19.59)	15/73 (20.55)	41/104 (39.42)	35/69 (50.72)	<0.0001
Height	117/378 (30.95)	7/41 (17.07)	18/93 (19.35)	13/72 (18.06)	39/103 (37.86)	40/69 (57.97)	<0.0001
Head circumference	99/363 (27.27)	5/40 (12.5)	11/91 (12.09)	12/68 (17.65)	33/99 (33.33)	38/65 (58.46)	<0.0001
Respiratory support							
Use of oxygen	39 (10.1.)	0 (0)	2 (2.06)	3 (4)	10 (9.62)	24 (34.78)	<0.0001
Ventilator care	4 (1.04)	0 (0)	0 (0)	0 (0)	1 (0.96)	3 (4.35)	0.0600
Medication for PHT	7 (1.81)	0 (0)	0 (0)	1 (1.33)	2 (1.92)	4 (5.8)	0.0861

CDD, cumulative dexamethasone dose; NDI, neurodevelopmental impairment; PHT, pulmonary hypertension. Values are expressed as number (%). *p*-values are calculated by one-way ANOVA or Kruskal–Wallis test for continuous variables and Chi-square test or Fisher’s exact test for categorical variables, depending on data distribution and sparsity.

**Table 5:** Associations of long-term growth and neurodevelopmental outcomes at corrected age of 18–24 months.

Variables	D0 (No Postnatal Corticosteroid Use, *n* = 41) ^a^	D1 (CDD < 2 mg/kg, *n* = 97) ^a^	D2 (2 ≤ CDD < 4 mg/kg, *n* = 75)	D3 (4 ≤ CDD < 8 mg/kg, *n* = 104) ^a^	D4 (CDD ≥ 8 mg/kg, *n* = 69) ^a^
Primary Outcome					
NDI or death	0.508 [0.104, 2.469]	1.228 [0.498, 3.025]	1 (Reference)	1.233 [0.525, 2.894]	3.550 [1.260, 10.004] *
Secondary Outcome					
Cerebral palsy	1.418 [0.069, 29.137]	0.447 [0.059, 3.377]	1	1.161 [0.262, 5.140]	1.587 [0.294, 8.569]
NDI	0.603 [0.117, 3.121]	1.353 [0.522, 3.504]	1	1.382 [0.567, 3.366]	3.479 [1.074, 11.268] *
Motor delay	0.479 [0.028, 8.155]	1.947 [0.541, 7.005]	1	1.600 [0.476, 5.381]	6.825 [1.689, 27.584] *
Mental delay	0.690 [0.132, 3.616]	1.558 [0.570, 4.257]	1	1.884 [0.731, 4.855]	4.263 [1.318, 13.787] *
Social delay	0.664 [0.038, 11.569]	1.119 [0.283, 4.420]	1	1.538 [0.475, 4.979]	2.727 [0.683, 10.891]
Failure to achieve catch-up growth (<10th percentile)	1.821 [0.419, 7.909]	1.424 [0.513, 3.949]	1	2.291 [0.895, 5.865]	4.077 [1.217, 13.656] *
Weight	0.966 [0.198, 4.710]	0.905 [0.311, 2.634]	1	1.966 [0.755, 5.118]	2.799 [0.879, 8.914]
Height	1.736 [0.394, 7.653]	1.077 [0.361, 3.217]	1	2.310 [0.866, 6.164]	3.915 [1.218, 12.582] *
Head circumference	1.697 [0.284, 10.140]	0.861 [0.243, 3.046]	1	1.821 [0.631, 5.255]	3.004 [0.846, 10.667]

CDD, cumulative dexamethasone dose; NDI, neurodevelopmental impairment. ^a^ Adjusted for gestational age, 1 min Apgar score, intraventricular hemorrhage (grade ≥ 3), periventricular leukomalacia, retinopathy of prematurity requiring laser therapy, bronchopulmonary dysplasia (≥moderate), necrotizing enterocolitis (stage ≥ 2), duration of parenteral nutrition, culture-proven sepsis, small for gestational age, and length of hospital stay. Values are expressed as adjusted odds ratio [95% confidence interval]. An asterisk (*) indicates pairwise comparisons with statistically significant *p*-values.

## Data Availability

All data generated or analyzed during this study are included in this published article. The datasets used and/or analyzed during the current study are available from the corresponding author on reasonable request.

## Supplementary Materials

The following supporting information can be downloaded at: https://www.mdpi.com/article/10.3390/jcm15135023/s1, Table S1: Postnatal Corticosteroid Use According to Gestational Age.

## Abbreviations

The following abbreviations are used in this manuscript:

BPD	Bronchopulmonary dysplasia
BSID	Bayley Scales of Infant and Toddler Development
CDD	Cumulative dexamethasone dose
CI	Confidence interval
CRIB	Clinical Risk Index for Babies
EPI	Extremely preterm infant
IQR	Interquartile range
K-DST	Korean Developmental Screening Test
NDI	Neurodevelopmental impairment
NICU	Neonatal intensive care unit
OR	Odds ratio
