# Supplementary material for: Threshold Effect of Cumulative Postnatal Corticosteroid Dose on Long-Term Outcomes in Extremely Preterm Infants
Source: J Clin Med. 2026 Jun 27;15(13):5023. doi: 10.3390/jcm15135023 (PMC13362759; doi:10.3390/jcm15135023)
Supplement: Supplementary file 1 [file jcm-15-05023-s001.zip › jcm-4312097-supplementary.pdf]

Supplementary Table S1. Postnatal Corticosteroid Use According to Gestational Age

**Variables**	**GA 22wks**	**GA 23wks**	**GA 24wks**	**GA 25wks**	**GA 26wks**	**GA 27wks**	**Total**	***p*-value**
n	18	72	104	116	106	102	518	
Hydrocortisone use, *n* (%)	18 (100)	72 (100)	92 (88.64)	98 (84.48)	81 (76.42)	52 (50.98)	413/518 (79.73)	<0.0001
Dexamethasone use, *n* (%)	14 (77.78)	49 (68.06)	72 (69.23)	73 (62.93)	63 (59.43)	41 (40.20)	312/518 (60.23)	0.2485
Prednisolone use, *n* (%)	7 (38.89)	17 (23.61)	30 (28.85)	34 (29.31)	19 (17.92)	18 (17.65)	125/518 (24.13)	0.3596
Any steroid use, *n* (%)	18 (100)	72 (100)	103 (99.04)	113 (97.41)	98 (92.45)	71 (69.61)	475/518 (91.7)	<0.0001
CDD, mg/kg (*n*=475)	5.8 [3.58, 8.06]	7.38 [3.67, 11.27]	5.22 [3.4, 10.21]	4.41 [2.1, 7.46]	2.7 [1.19, 5.43]	2.39 [1.12, 4.8]	4.24 [2, 8.06]	<0.0001
Cumulative hydrocortisone, mg/kg (*n*=413)	78.17 [25.95, 117.54]	72.42 [33.52, 137.77]	44.15 [19.53, 121.28]	34.67 [14.07, 63.78]	28.38 [14.53, 68.86]	20.56 [8.95, 48.03]	37.99 [15.62, 85.44]	<0.0001
Cumulative dexamethasone, mg/kg (*n*=312)	1.35 [0.72, 2.67]	3.03 [1.63, 6.93]	4.31 [1.9, 6.97]	3.9 [2.67, 6.34]	3 [0.89, 4.81]	2.39 [0.9, 4.24]	3.42 [1.24, 6.2]	0.0001
Cumulative prednisolone, mg/kg (*n*=125)	17.09 [16.08, 20.33]	27.96 [15.37, 54.72]	14.46 [10.13, 17.34]	16.78 [14.63, 30.89]	16.32 [15.74, 23]	17.62 [14.85, 27.02]	16.68 [14.38, 27.96]	0.0537

GA, gestational age; CDD, cumulative dexamethasone dose. Values are expressed as median [IQR] or number (%).
